# High Fat Diet Induces Adhesion of Platelets to Endothelium in Two Models of Dyslipidemia

**DOI:** 10.1155/2014/591270

**Published:** 2014-09-28

**Authors:** Jaime Gonzalez, Wendy Donoso, Natalia Díaz, María Eliana Albornoz, Ricardo Huilcaman, Erik Morales, Rodrigo Moore-Carrasco

**Affiliations:** ^1^Departamento de Bioquímica Clínica e Inmunohematología, Facultad de Ciencias de la Salud, Universidad de Talca, P.O. Box 747, Talca, Chile; ^2^Departamento de Estomatología, Facultad de Ciencias de la Salud, Universidad de Talca, Avenida Lircay s/n, Talca, Chile; ^3^Departamento de Ciencias Básicas Biomédicas, Facultad de Ciencias de la Salud, Universidad de Talca, Avenida Lircay s/n, Talca, Chile; ^4^Facultad de Medicina, Universidad Católica del Maule, Avenida San Miguel 3605, 3480112 Talca, Chile; ^5^Centro de Estudios en Alimentos Procesados (CEAP), Conicyt-Regional, Gore Maule, R09I2001, Avenida San Miguel 3425, 3480137 Talca, Chile; ^6^Programa Investigación de Excelencia Interdisciplinario en Envejecimiento Saludable PIEI-ES, Universidad de Talca, Avenida Lircay s/n, Talca, Chile

## Abstract

Cardiovascular diseases (CVD) represent about 30% of all global deaths. It is currently accepted that, in the atherogenic process, platelets play an important role, contributing to endothelial activation and modulation of the inflammatory phenomenon, promoting the beginning and formation of lesions and their subsequent thrombotic complications. The objective of the present work was to study using immunohistochemistry, the presence of platelets, monocytes/macrophages, and cell adhesion molecules (CD61, CD163, and CD54), in two stages of the atheromatous process. CF-1 mice fed a fat diet were used to obtain early stages of atheromatous process, denominated early stage of atherosclerosis, and ApoE^−/−^ mice fed a fat diet were used to observe advanced stages of atherosclerosis. The CF-1 mice model presented immunostaining on endothelial surface for all three markers studied; the advanced atherosclerosis model in ApoE^−/−^ mice also presented granular immunostaining on lesion thickness, for the same markers. These results suggest that platelets participate in atheromatous process from early stages to advance d stages. High fat diet induces adhesion of platelets to endothelial cells *in vivo*. These findings support studying the participation of platelets in the formation of atheromatous plate.

## 1. Introduction

Cardiovascular diseases (CVD) are the leading cause of death among adult population in most countries [1], in which the most common cause is the atheroma formation in arteries [2]. Atherosclerosis is a disease of multifactorial etiology and it is triggered by systemic and local factors, which finally cause vascular dysfunction. Hypercholesterolemia and especially high concentrations of low density lipoproteins (LDL) are a significant risk factor for premature appearance of atherosclerosis and ischemic heart disease [3].

A number of different cellular components perform an important role in the pathogenesis of atherosclerosis. It is well established that endothelial cells, as well as intimal smooth muscle cells, are involved [4]. On the other hand, monocytes and macrophages are crucial in early stages [5]. Currently, there are reports which accept that platelets play a crucial role in plaque formation [6]. Over the last years, it has been demonstrated that platelets participate not only in thrombotic complications of atheromatous lesion, but also in the initiation and progression of the plaque [7], contributing to the endothelial activation and modulation of inflammatory process, which favor the initiation and formation of atheromatous lesions and their subsequent thrombotic complications [8]. Predominant involvement of platelets in atherosclerotic lesion formation occurs through endothelial activation [9]. Platelet adhesion to endothelial surface generates signals that recruit monocytes to sites of inflammation [10], inducing a proatherogenic phenotype characterized by a major adhesion, chemotaxis, and proteolytic activity [11, 12].

The study of the atherosclerotic process in animals is complex with a multifactorial mechanism; however, the use of high fat diets in mice is a widely used strategy. Previously, our study group characterized a mouse model of metabolic syndrome (MS) in CF1 mice, fed a diet with 22% fat; these mice developed alterations in lipid and glucose metabolism and endothelial dysfunction [13].

Apolipoprotein E deficient mouse (ApoE^−/−^) is a well-established model to study atherogenesis [14] because it is unable to perform the reverse cholesterol transport, which implies an accumulation of lipid elements at luminal level in blood vessels. A diet rich in fat contributes to early atherosclerotic lesion development, as foam cell formation was observed at week 10, intermediate lesions with foam cell content and smooth muscle cells at week 15, and fibrous plaques development at week 20 [15].

## 2. Material and Methods

### 2.1. Animal Models

#### 2.1.1. CF1 Mice: A Model of Early Stage of Atherosclerosis

Two groups of four CF-1 mice obtained from the Public Health Institute of Chile (ISP) were used. The first group was fed a fat diet (FD) and the second group a normal diet (ND), using Champion mouse food, for a period of 40 days [13].

#### 2.1.2. ApoE Knockout Mice: A Model of Advanced Atherosclerosis

Two experimental groups with six individuals each were established; they were constituted by ApoE^−/−^ mice (donated by Dr. Atilio Rigotti from Pontificia Universidad Católica de Chile), fed ND and FD for 20 weeks.

#### 2.1.3. Maintenance and Ethical Considerations

Animals used for experimentation were maintained in standard environmental conditions: temperature was 22 ± 2°C, photoperiod was 12 hours of light, 12 hours of darkness, and they had free access to water and food.

For experimental procedures, the protocol of the Manual of Care and Maintenance of Animals for Experimentation of the National Commission for Scientific and Technological Research of Chile (CONICYT) was followed. All protocols and procedures were approved by the Bioethics Committee from Universidad de Talca.

#### 2.1.4. Immunohistochemistry

Tissues of ApoE^−/−^ mouse aortas and CF-1 mouse coronary arteries were used for immunohistochemical studies. Samples were fixed in formaldehyde 4% and 0.075 M sodium phosphate buffer, pH 7.3, and paraffin-embedded. Later, they were cut in sections of 5 *μ*m thickness and then deparaffinized and hydrated.

They were subjected to antigen retrieval with sodium citrate buffer 0.01 M, pH 6, and endogenous peroxidases were inhibited with hydrogen peroxide 3%. Later, Mouse monoclonal primary antibodies, (Novocastra, UK) anti-CD61 (to platelets), anti-CD163 (to monocyte/macrophages), and anti-CD54 (ICAM-1), were applied in concentrations 1/25, 1/50, and 1/25, respectively, to each of the analyzed samples and incubated for 30 min at 37°C. The VECTOR M.O.M. Immunodetection Kit (Vector Laboratories, USA) was used as a detection system following the manufacturer's instructions and diaminobenzidine (DAB) ImPACT DABPeroxidase Substrate (Vector Laboratories, USA) was used to reveal the presence of the protein of interest. Then, a nuclear contrast was done with Harris haematoxylin and samples were assembled in a resinous medium. Simultaneously, histochemical staining of hematoxylin-eosine (H&E) and Masson's trichrome was done according to Kiernan, 2008 [16], for histopathological analysis.

### 2.2. Statistical Analysis

The statistical analysis of the results was done by the analysis of variance (ANOVA) using Graphpad software. The results are presented as average and standard error of the mean (SEM). The significance of variables was evaluated using a confidence interval of 95%.

## 3. Results

First, we prepared diets supplemented with fat.  Table 1 shows the analysis of biochemical parameters and initial and final weight in each group of mice; ND can be homologated to a human diet since they have similar percentage of the different components, while the FD have higher content of fat (25%) which can be homologated with occidental diets and fast food.

### 3.1. Model of Early Stage of Atherosclerosis

First, we tested high fat diet in CF1 mice to induce metabolic alterations and endothelial dysfunction [13].

**Figure 1:**
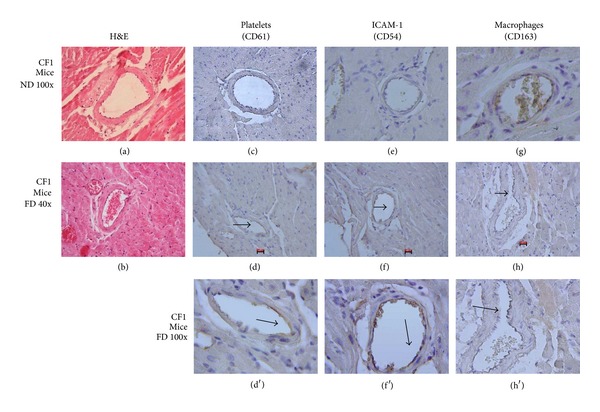
Coronary of CF1 mice a SM Model. ((a), (b)) Histological sections of CF1 mouse coronary artery ND and FD, stained with H&E (100x). (c) Histological section of coronary artery of CF1 mouse ND. ((d), (d′)) Histological sections of coronary artery of CF1 mouse FD. Immunohistochemistry technique for platelets (CD61), 1/25. (e) Histological section of coronary artery of CF1 mouse ND. ((f), (f′)) Histological sections of coronary artery of CF1 mouse FD. Immunohistochemistry technique for ICAM-1, 1/25. (g) Histologic section of coronary artery of CF1 mouse ND. ((h), (h′)) Histological sections of coronary artery of CF1 mouse FD. Immunohistochemistry technique for monocytes/macrophages (CD163), 1/25. Scale bar: 20 *μ*m.

Figure 1 includes images of histological sections of CF-1 mouse coronary arteries fed with ND and FD. Panels (a) and (b) show histological sections stained with H&E. A conserved histoarchitecture is observed but atherosclerotic lesions are not observed. Images (c), (d), and (d′) correspond to histological sections stained with immunohistochemistry technique for platelets. Image (c) shows conserved histoarchitecture and immunostaining absence. Image (d) shows conserved histoarchitecture and moderate immunostaining of endothelial surface. Images (e), (f), and (f′) correspond to histological staining with immunohistochemical technique for ICAM-I. Image (e) presents conserved histoarchitecture and immunostaining absence and image (f) shows conserved histoarchitecture and moderate immunostaining of endothelial surface. Images (g), (h), and (h′) correspond to histological sections, stained with monocyte/macrophages immunohistochemical technique. Image (g) presents conserved histoarchitecture and immunostaining absence. Image (h) shows conserved histoarchitecture and moderate immunostaining of endothelial surface. 40x and 100x magnification were used in all sections.

### 3.2. Model of Advanced Atherosclerosis

**Figure 2:**
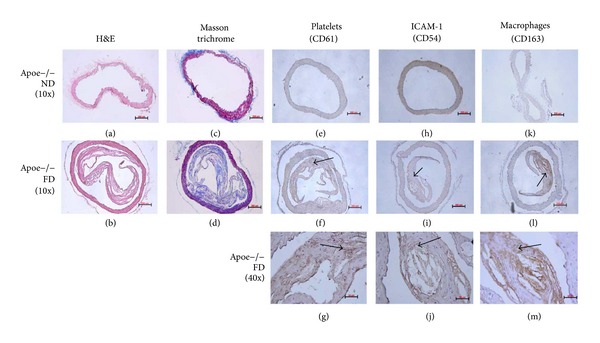
APOE^−/−^ mice. Advanced atherosclerosis model. ((a), (c)) Histological section of APOE^−/−^ mice fed with ND. ((b), (d)) Histological section of ApoE^−/−^ mice fed with FD. H&E staining and Masson trichrome. (e) Histological section of ApoE^−/−^ mice fed with ND. ((f), (g)) Histological sections of ApoE^−/−^ mice fed with FD. Immunohistochemistry for platelets (CD61), 1/25. (h) Histological section of ApoE^−/−^ mice fed with ND. ((i), (j)) Histological sections of ApoE^−/−^ mice fed with FD. Immunohistochemistry for ICAM-1, 1/25. (k) Histological section of ApoE^−/−^ mice fed with ND. ((l), (m)) Histological sections of ApoE^−/−^ mice fed with FD. Immunohistochemistry for ICAM-1, 1/25. Scale bar: 50 and 200 *μ*m.

In the next step, we tested our diets in ApoE^−/−^ mice.  Figure 2 includes images of histological sections of aorta artery of ApoE^−/−^ mice fed with ND and FD. Images (a) and (b) correspond to H&E staining, 10x magnification. In image (a) a conserved histoarchitecture is observed, with no apparent lesions. In Figure 2(b) histoarchitecture distortion is observed, characterized by irregular thickening of the intima layer, partially detached, with apparently fibrous foci and optically empty spaces. Panels (c) and (d) correspond to Masson's trichrome staining, 10x magnification. Image (c) shows conserved histoarchitecture with no apparent lesions. Image (d) highlights collagenous fibrosis foci and histoarchitectural distortion. Image (e) corresponds to histological sections of ApoE^−/−^ mouse fed with ND. A conserved histoarchitecture and immunostaining absence were observed. Images (f) and (g) correspond to histological sections stained with immunohistochemical technique for platelets, 10x and 40x magnification. A histoarchitecture distortion focus was observed characterized by irregular thickening of the intima layer, partially detached, with apparently fibrous foci and optically empty spaces. It highlights moderate immunostaining of endothelial surface and granular in lesion thickness. Image (h) corresponds to histological sections of ApoE^−/−^ mouse fed with ND, 10x magnification. A conserved histoarchitecture and immunostaining absence are observed. Images (i) and (j) correspond to histological sections stained with immunohistochemical technique for ICAM-I, 10x and 40x magnification. A histoarchitecture distortion focus was observed characterized by irregular thickening of the intima layer, partially detached, with apparently fibrous foci and optically empty spaces. Figures 2(i) and 2(j) highlights moderate immunostaining at endothelial level.

Image (k) corresponds to histological sections of ApoE^−/−^ mouse fed with ND, 10x magnification. A conserved histoarchitecture and immunostaining absence are observed. Images (l) and (m) correspond to histological sections stained with immunohistochemical technique for monocyte/macrophages, 10x and 40x magnification. Histoarchitecture distortion is observed characterized by irregular thickening of the intima layer, partially detached, with apparently fibrous foci and optically empty spaces. It highlights intense granular immunostaining in lesion thickness and adjacent to optically empty spaces (lipid core).

## 4. Discussion

CVD are the main cause of morbi-mortality in most occidental countries. In ischaemic CVD, atheromatous plate formation is a critical point; so, the physiopathology knowledge of this disease is very important. In this sense, the standardization of animal models developing different stages of atherothrombotic process, as well as the use of techniques which allow visualizing these changes, is of great help to increase the knowledge of the complex process of atherogenesis. Due to the previous fact, the objective of this work was to study through immunochemistry the participation of endothelium, monocyte/macrophage, and platelets in a metabolic syndrome (MS) model obtained in CF-1 mice and in an advanced atherosclerosis model obtained in ApoE^−/−^ mice. This allows having a global vision of the main actors in different stages of atherothrombosis physiopathology.

A model of CF-1 mice fed with FD that develops metabolic alterations like MS [13] was used for the study of the initial stage of atherosclerotic process, with primary stages of endothelial dysfunction. In physiological conditions, endothelium presents a surface with antithrombogenic properties [17] through which the exchange of numerous substances between blood and tissues is produced; it controls the vascular tone and the transit of inflammatory cells towards vascular wall. An early stage of atherosclerotic process is the development of the endothelial dysfunction, characterized, among other aspects, by the growing expression of cell adhesion molecules such as VCAM-I, E-selectin, and ICAM-I, allowing the binding of other cell types, a process that increases as the disease progresses. In the MS murine model, a slight positivity for ICAM-I expression was observed (Figure 1), characteristic at this early stage of endothelial dysfunction, in which this molecule is overexpressed in atherosclerotic inflammatory processes [18], validating our model as an endothelial damage promoter.

It is currently known that platelets can internalize oxidized low density lipoprotein (Ox-LDL) in a dyslipidaemia scenario and have the capacity to join endothelium with no need of denudation [9, 19, 20]. This was observed in the MS model, since slight positivity for platelets was visualized. This result agrees with those obtained in previous works mainly carried out* in vitro* and* in vivo* in ApoE^−/−^ models [9, 21]; however, this is the first time that was proved through immunohistochemical technique in a CF-1 mouse model with MS. The presence of monocytes/macrophages was also studied, observing slight positivity (Figure 1); this ratifies the endothelial dysfunction model in which there is increase of adhesion molecules such as ICAM-I and P-selectin among others [9], allowing the binding of platelets and monocytes. With the results obtained in the MS mice model it might be postulated that high cholesterol levels alters endothelial function (adhesion molecule increase) and platelets join the dysfunctional endothelium together with monocytes in coronary artery of CF-1 mice [22, 23].

An advanced atherosclerosis model was used for the study of more advanced stages in the atherosclerotic process. The ApoE deficient mouse is one of the best models as it provides great information, since these animals develop hypercholesterolemia spontaneously, with a cholesterol level up to five times higher than normal mice [24], developing atherosclerosis in a short period of time. This allows evaluating different molecules and cells that participate in physiopathology of atherosclerosis, as well as the influence of diet in such process [25]. In this work, the development of an atheromatous plaque in aorta artery of group FD mice was observed (Figure 2) but not so in the rest of the experimental group visualized with H&E and Masson's trichrome, which agrees with what Ni et al. described in experimental groups subjected to a high fat diet and periods of stress [26].

Over the last years there is evidence that platelets as well as erythrocytes can penetrate the plaque through angiogenic capillary breakages [27]. In advanced atherosclerosis model, in ApoE^−/−^ mice, subendothelial infiltration of monocytes/macrophages and platelets was observed, evidenced by a positivity for CD163 and CD61 immunolabelling, in subendothelial space and in lipid accumulation sectors (Figure 2). These results reaffirm the thesis of the interaction between platelets and macrophages, mainly in the atherosclerotic lesion site promoting an optimum scenario that might encourage the platelet contribution in foam cell formation [12]. There are* in vitro* studies demonstrating that platelet is involved in the accumulation of oxidized lipids by macrophages and subsequent transformation to foam cells [20, 28–30]. Platelets can accumulate lipids in a hypercholesterolemic environment [31], reaffirming this observation. There are* in vitro* studies in which platelets are able to contribute their lipid content to murine monocytes favoring foam cell formation, after their phagocytosis [32]. For this phenomenon to take place, macrophage and platelet must be in intimate contact, an event demonstrated* in vivo* in our results showing platelets and macrophages in contact inside the plaque. This adds up to the platelet mobilization capacity through a permeable membrane, as well as through the activated endothelium using chemoattractant molecules which are present in vascular inflammatory events [33]; other researchers proved that platelets go through the intestinal epithelium to participate in inflammatory processes and this step would be facilitated by comigration with polymorphonuclear leukocytes (PMN) [34], proposing novel capacities to platelets in inflammatory events such as atherosclerosis. Our results show that platelets are present in atheromatous plaques obtained* in vivo* and can interact with macrophages in lipid core, producing different molecules that favor the development of atherosclerotic plaque through this interaction [30, 35].

However, this cannot be assured with the techniques used in this study, but their presence in the lesion site might favor such process.

Adhesion molecules are key in the cellular recruiting to the inside of the vessel wall, expressing themselves on endothelial cells activated in response to inflammatory cytokines, as IL-1*β* and TNF-*α* parallel to leukocyte migration [36] clearly evidenced in the atheromatous process, where some researchers relate ICAM-I overexpression with events involving endothelial damage [37]. This was proved in the advanced atherosclerosis model in ApoE^−/−^ mice, finding strong positivity not only in endothelial cells but also in the subendothelial space, correlating with the progression of the disease as well as the infiltration level of macrophages and platelets in the lesion site. These results ratify the importance given by different authors in the study of ICAM-I as an early marker of atherosclerosis [38]. It would be interesting to know the real participation of high cholesterol concentrations added to the inflammatory response, obtained with the model of ApoE^−/−^ mouse, in overexpression of this adhesion molecule, since it has been described that atorvastatin, together with reducing cholesterol, also decreases plasma soluble ICAM-I concentrations [39].

## 5. Conclusion

Using immunostaining, it can be observed that platelets participate from early stages in the atheromatous process, initially adhering to the endothelium, which increases as the disease progresses, until infiltrating markedly the sites where there is a plaque and an inflammatory process. This is probably favored by the increase of the expression of adhesion molecules at endothelial level as well as high lipid concentrations that might modify directly the platelets increasing their transmigration. A narrow contact with macrophages could also be evidenced mainly in sites where there are fatty deposits, which would encourage interaction between both. In future studies, it would be interesting to test whether platelets have the capacity to form foam cells directly. This would allow giving relevant information useful for the implementation of a new therapeutic target in the fight against CVD.

## Figures and Tables

**Table 1:** Biochemical parameters, initial and final weight in different groups of ApoE^−/−^ and CF-1 mice subjected to different diets.

Biochemical component	ApoE^−/−^ ND (4)	ApoE^−/−^ FD (4)	CF-1 ND (4)	CF-1 FD (4)
Total cholesterol (mg/dL)	318 ± 34	533 ± 77∗	90 ± 3	178 ± 15∗
Triglyceridemia (mg/dL)	141 ± 8	185 ± 21	95 ± 9	223 ± 38∗
Glycemia (mg/dL)	161 ± 29	324 ± 64∗	130 ± 6	283 ± 19∗
Initial weight (g)	32.8 ± 1.9	35.1 ± 0.6	27.6 ± 0.4	28.7 ± 0.2
Final weight (g)	35.1 ± 1.2	43.4 ± 4.3∗	40.4 ± 0.8	44.7 ± 2.6∗

ND: normal diet; FD: fat diet.

The results are expressed as the mean ± SEM. Statistical significance: ∗significant at *P* < 0.05.
